# Enhanced Unipolar Resistive Switching Characteristics of Hf_0.5_Zr_0.5_O_2_ Thin Films with High ON/OFF Ratio

**DOI:** 10.3390/ma10030322

**Published:** 2017-03-22

**Authors:** Zhipeng Wu, Jun Zhu

**Affiliations:** State Key Laboratory of Electronic Thin Films and Integrated Devices, University of Electronics Science and Technology of China, Chengdu 610054, China; wuzhiroc@live.com

**Keywords:** Hf_0.5_Zr_0.5_O_2_ thin films, resistive switching, RRAM, PLD

## Abstract

A metal–insulator–metal structure resistive switching device based on H_0.5_Z_0.5_O_2_ (HZO) thin film deposited by pulse laser deposition (PLD) has been investigated for resistive random access memory (RRAM) applications. The devices demonstrated bistable and reproducible unipolar resistive switching (RS) behaviors with an extremely high OFF/ON ratio over 5400. The retention property had no degradation at 6 × 10^4^ s. The current–voltage characteristics of the HZO samples showed a Schottky emission conduction in the high voltage region (V_reset_ < V < V_set_), while at the low voltage region (V < V_reset_), the ohmic contact and space charge limited conduction (SCLC) are suggested to be responsible for the low and high resistance states, respectively. Combined with the conductance mechanism, the RS behaviors are attributed to joule heating and redox reactions in the HZO thin film induced by the external electron injection.

## 1. Introduction

Resistive switching (RS) devices based on various binary transition metal oxides, such as TiO_2_ [1,2,3], NiO_x_ [4,5], ZnO [6,7], HfO_2_ [8,9,10,11,12], ZrO_2_ [13,14,15,16], etc., have been extensively studied for their great potential as excellent substitutes in non-volatile memory applications. Such kinds of devices are commonly referred to as resistive random access memory (RRAM). The devices usually show bistable resistance state, and one can switch from a conductive ON state (low resistance state, LRS) to a less conductive OFF state (high resistance state, HRS) by applying electrical bias to the device, and vice versa. The switching operation is called unipolar when the switching procedure does not depend on the polarity of the write voltage. In contrast, the operation is called bipolar when the set to ON state occurs at one voltage polarity and the reset to OFF state on reversed voltage polarity [17,18]. The simple metal/insulator/metal (MIM) structure of these devices makes them quite easy to fabricate and integrate with other processes. In recent years, HfO_2_- and ZrO_2_-based thin films have been extensively used as promising “*high-k*” substitutes for integrated oxide gate dielectrics driven by the tendency of down-scale in complementary metal–oxide–semiconductor (CMOS) applications [19]. This makes it possible to integrate the HfO_2_- and ZrO_2_-based RS devices with CMOS technology. However, most recent studies show that both HfO_2_ and ZrO_2_ have some disadvantages in resistive switching memory applications. HfO_2_-based thin films usually demonstrate a bipolar resistive switching behavior and need a negative polarity power supply support, while ZrO_2_-based thin films resistive switching devices typically have a poor uniformity behavior, which remains an obstacle for their application. Moreover, both RS behaviors operate with a relatively small OFF/ON ratio of the HfO_2_ or ZrO_2_ thin films. Wu [8] studied the HfO_2_/ITO/Invar structure with conductive atomic force microscopy, and a ratio of about 100 was revealed with this unipolar RS structure. A Ti/ZrO_2_/Pt structure was fabricated by Zhang [16], and an OFF/ON ratio of about 24 was reported. Sharath [9] investigated the RS behaviors in engineered oxygen-deficient HfO_2-x_ thin films with various thicknesses. It was suggested that both stoichiometric HfO_2_ and nonstoichiometric HfO_2-x_ show bipolar RS behaviors with a relatively small OFF/ON ratio of approximately 5. Ag/HfO_2_/ITO and TiN/HfO_2_/ITO structures were studied by Ramadoss [11] and Ye [12], respectively. Both structures presented a bipolar RS behavior with OFF/ON ratios not over 10. Meanwhile, the essential negative power in bipolar RS devices has also increased the complexity of the integrated system [20]. To improve the applications in RRAM and integrated systems, a promising unipolar RS material with high OFF/ON ratio is particularly important. Several groups [20,21,22] tried to use the HfO_2_/ZrO_2_ bilayer structure to improve the OFF/ON ratio and stability of the device, and better results were observed. This makes us believe that the HfO_2_-ZrO_2_ solution might provide a better performance.

In this letter, the RS characteristics of a H_0.5_Z_0.5_O_2_ (HZO) thin film prepared by pulse laser deposition (PLD) for RRAM application have been investigated. This HZO thin film reveals a bistable RS property with extremely high OFF/ON ratio. To understand the RS mechanism in this film, the current conductive mechanism is also discussed.

## 2. Materials and Methods

The HZO films were deposited on Pt/Ti/SiO_2_/Si substrates by PLD with a ceramic Hf_0.5_Zr_0.5_O_2_ target synthesized by solid phase sintering with high purity HfO_2_ and ZrO_2_ powder. During deposition, the energy of the laser beam was fixed at 2 J/cm^2^. The thickness of the HZO thin film was about 50 nm. For electrical measurement, the Ni (40 nm)/Au (150 nm) top electrodes with a diameter of 190 μm were deposited by electron beam evaporation using a shadow mask to form the metal–insulator–metal (MIM) sandwich structure. The current–voltage (*I-V*) characteristics of the MIM structures were measured using an Agilent 4156C precision semiconductor parameter analyzer (Agilent Technologies, Santa Clara, CA, USA) at room temperature with the humidity less than 30%. RS measurement was performed by applying a DC voltage sweep with the shape of a staircase to the top electrode while the bottom electrode was grounded. For the endurance testing measurement, we used the staircase sweep measurement mode with a delay time of 1 s. For the retention testing measurement, we read the current at different times with an applied pulse voltage of 0.2 V on the devices at 85 °C. The current compliance of 5 mA was used for the forming and set process. Chemical valence states of elements in the film were analyzed with Al Kα source XSAM800 X-ray photoelectron spectroscopy (XPS, Kratos Analytical, Manchester, UK).

## 3. Results and Discussion

**Figure 1:**
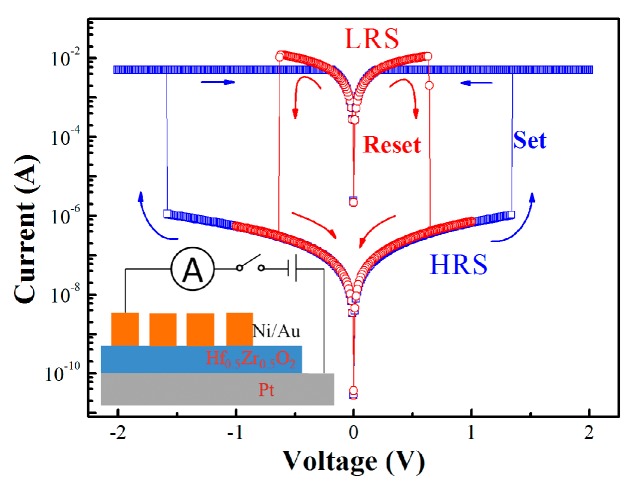
Typical unipolar resistive switching (RS) current–voltage curves for Au/Ni/HZO/Pt device. The inset shows the scheme of metal/insulator/metal (MIM) structure. HRS: high resistance state; LRS: low resistance state.

Figure 1 shows the typical *I-V* characteristics of the HZO samples taken from the cycles test. Before that, the forming process was taken by applying a sweep voltage from 0 to 5 V with compliance current at 100 µA. The unipolar switching behavior is revealed in this figure. When a positive electrical stress was applied to the HRS sample, the current increased to the compliance current abruptly, while the device switched to LRS. This is defined as the set process, and the V_set_ main range is from 1.8 to 2.2 V. On the other hand, the reset process was also achieved by sweeping the voltage from zero to the V_reset_, which mainly ranges from 0.7 to 0.9 V to switch the device from LRS to HRS. The current switching phenomenon is also observed at negative voltage sweeping, confirming the unipolar RS in the HZO samples [17].

**Figure 2:**
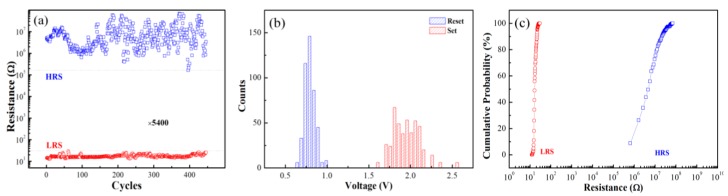
(**a**) Endurance characteristics of the H_0.5_Z_0.5_O_2_ (HZO) device; the currents are read at 0.2 V; (**b**) Set and Reset voltage distribution taken from the endurance cycles; (**c**) Distribution of the resistance at HRS and LRS taken from the endurance cycles.

**Figure 3:**
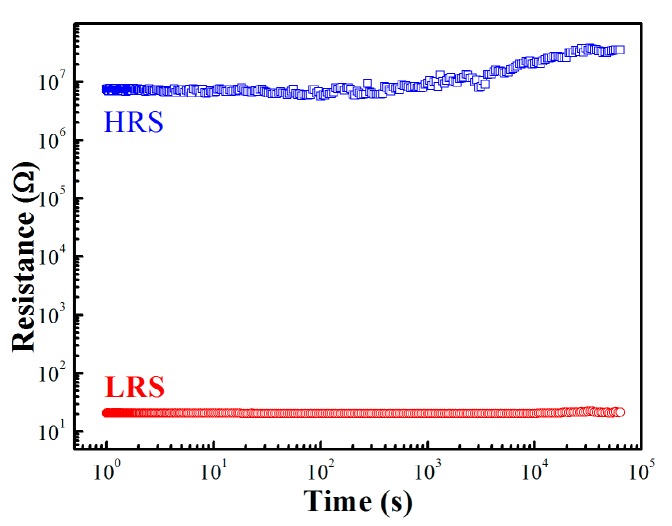
Retention characteristics of the HZO device; the currents are read at 0.2 V.

Figure 2a shows the endurance characteristic of the MIM structure with the resistance of HRS and LRS as a function of the number of switching cycles measured at the reading voltage of 0.2 V. During the progress of successive RS up to 450 cycles, the OFF/ON ratio was at least 5400 with no obvious degradation observed—an OFF/ON ratio of 10 is a sufficient separation for the nonvolatile memory application [23], which means that our HZO sample is far beyond the basic requirement. Figure 2b demonstrates the distributions of Set/Reset voltage taken from the endurance cycles. The stander deviation of Set and Reset voltages are 0.17 and 0.06, respectively. Figure 2c shows the cumulative probability distribution of the resistance at LRS and HRS. As shown in Figure 2b,c, the HZO-based RS device demonstrates good uniformity. The retention property is another significant characteristic of the nonvolatile memory device [17,23], and demonstrates the ability of a device to retain information after writing the stored data. Figure 3 shows the retention characteristic of the HZO sample. During the stress progress, a small voltage of 0.2 V was applied to read the resistance state of the sample. The resistance of both HRS and LRS conditions showed no degradation over 6 × 10^4^ s. All of these electrical properties indicate that the HZO thin film is an excellent RS material that has a high potential for RRAM application.

**Figure 4:**
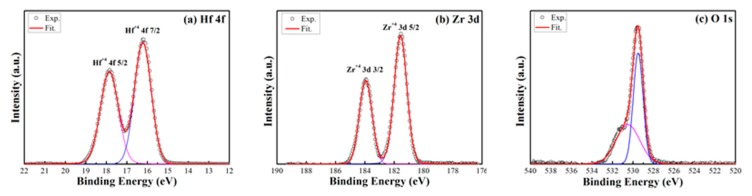
X-ray photoelectron spectroscopy (XPS) spectra of (**a**) Hf; (**b**) Zr; and (**c**) O in the original HZO sample.

XPS results are shown in Figure 4 to investigate the chemical states of Hf, Zr, and O in the initial HZO film. Before the XPS analysis, the original thin film without top electrode was pre sputter-etched with Ar ions to remove the surface contaminants. All spectra were calibrated with a C 1s binding energy located at 248.6 eV. Shirley backgrounds were removed from all spectra, and the oxide-related peaks were fitted with Gaussian–Lorentzian peak functions. The XPS results displayed in Figure 4a show that the 4f 5/2 and 4f 7/2 peaks of Hf are with binding energies of 17.85 and 16.21 eV. The intensity ratio of the components of the Hf 4f doublet and the spin–orbit splitting are 0.75 and 1.64 eV, respectively. The sample could be fitted with a single peak attributed to the Hf^4+^ oxidation state [9] with a full width at half maximum (FWHM) equal to 0.95 eV. Figure 4b shows that Zr 3d5/2 and Zr 3d3/2 peaks have binding energies of 181.57 and 183.94 eV, respectively. The energy interval of 2.37 eV between these two peaks represents the oxide state of Zr^4+^ [14]. The non-symmetric oxygen O 1s peak in Figure 4c can been fitted into two peaks with the binding energies of 529.5 and 530.5 eV. The higher peak with a FWHM of 1.13 eV is for O 1s bonded to the Hf^4+^, while the lower one with a FWHM of 2.73 eV is bonded to the Zr^4+^. The concentration ratio of Zr/Hf is 0.86, comparable to the original ratio of 1. This means that these are only hafnia and zirconia two oxide in the initial HZO sample. Hence, the XPS results demonstrate that there is no elemental state Hf or Zr in the sample and both transition metal elements are bonded to the oxygen. The oxide forms of these metallic constituents are those of HZOs, which have been reported to present bipolar RS behavior independently [8,9,13].

**Figure 5:**
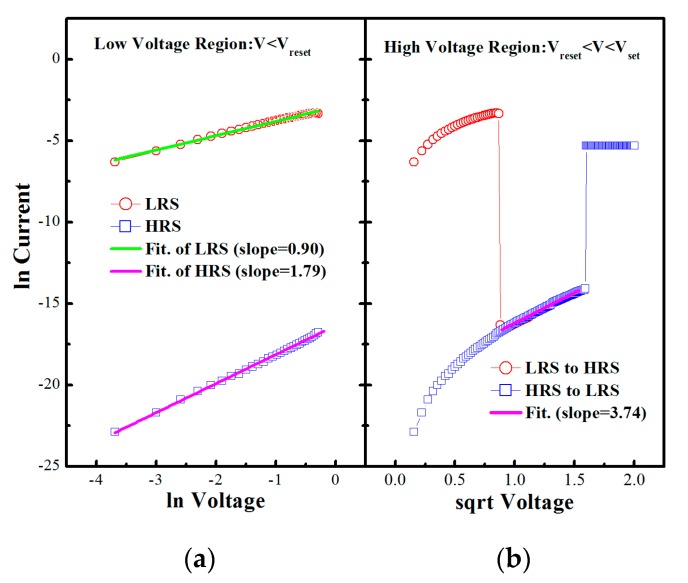
Current–voltage curve fitting of the HZO device in (**a**) log vs. log scale and (**b**) log vs. square root scale.

The typical current–voltage curves of the Au/Ni/HZO/Pt MIM structure in the low voltage region (V < V_reset_) are plotted in log vs. log scale in Figure 5a. The current–voltage curves fitting for both LRS and HRS are straight lines, while a slope = 0.9 is present at LRS and slope = 1.79 is found at HRS. This indicates an ohmic conduction mechanism considered because of the formation of a conductive filament during the set procedure. However, for the HRS, the slope is approximately 2, which suggests that the conduction at HRS is presumably dominated by the trap control space charge limited conduction (SCLC) mechanism [7].

In the high voltage regions (V_reset_ < V < V_set_), the current–voltage curves are plotted in log vs. square root scale, as shown in Figure 5b. It is natural to suggest that the Ni/HZO/Pt structure has a Schottky nature because the Schottky equation is widely used in the leakage current analysis of the metal/semiconductor structures [14]. Note that the current–voltage curves have a linear relationship between log current and square root voltage, which indicates a Schottky emission conductive mechanism in this voltage region, while the voltage dependence of this mechanism is

(1) $$J=AT^{2}\exp(-\varphi /kT)\exp\left[\beta_{1}{\left(V/d\right)}^{1/2}\right]$$

where *J* is current density, V is applied voltage, and *T* is temperature. Hence, it appears that the current–voltage characteristics of the HZO thin film are governed by the Schottky conduction mechanism in the high voltage region V_reset_ < V < V_set_.

According to the analysis results given above, the switching of the initial HZO film to LRS is due to the forming process, which is accompanied by the soft breakdown. For the reset process, the resistance increased suddenly, which indicates that the filamentary conducting paths might have been ruptured. A joule heating effect caused by the external current is considered for the rupture of filaments. By increasing the voltage to V_reset_, the high current flow through many filaments heated up the film, which induced a simultaneous rupture of the filaments, and the HRS was achieved. For the set process, because the sample is composed of stoichiometric ZrO_2_ and HfO_2_, it is impossible to form conducting filaments by oxygen vacancies (V_O_S). This suggests that some reactions might take place in the sample during the set process. It is reasonable to suggest that an oxygen-deficient region can be formed during the electroforming process. Transition metal cations accommodate this deficiency by trapping electrons injected from the cathodes [17]. In the case of HZO,

(2) $$ne^{-}+M^{4+}\to M^{(4-n)+}$$

where *M* stands for Hf and Zr transition metal elements. At the anode, the oxidation reaction may lead to the evolution of oxygen gas, according to

(3) $$O_{O}\to \;V_{o}^{\prime \prime }+2e^{-}+1/2O_{2},$$

where $V_{o}^{\prime \prime }$ denotes oxygen vacanciy with a double positive charge with respect to the regular lattice and *O_O_* represents an oxygen ion on a regular site. The consequent joule heating effect significantly increases the local generation of V_O_S at the anodic interface and their drift and diffusion toward the cathode. The generated abundant V_O_S move to the location where electron injection occurs due to the electrostatic force and then accumulate there, leading to an M_n_O_2n-1_ filament. The forming process is complete after the filament connects the cathode and anode, and the LRS is achieved. During the set process, the incomplete filament that ruptured at the reset process is modified by joule heating. The external current flow warms up the thin film, abundant generated V_O_S are moved to the virtual electrode (remaining incomplete filament), driven by electrostatic force, until the filament is completed as in the forming process. Because of the compliance current, a conductive filament with a controlled resistance is formed composed of the sub-oxides [24,25]. Considering the details of the Set procedure, the ruptured filament forms incompletely, and takes the role of virtual electrode. Additional filaments could be successively formed near the first filament by local joule heating and electron injection due to a high current density [4]. Normally, multiple conductive filaments could lead to several HRS regions by applying different voltages, which is in accordance with the experimental data (not shown here). Therefore, the observed enhanced bistable resistance switching in HZO samples can be attributed to the joule heat and the redox reactions induced by the external electron injection.

## 4. Conclusions

In conclusion, high OFF/ON ratio RS properties of the HZO thin film deposited by PLD have been investigated for RRAM applications. Each MIM cell demonstrated bistable and reproducible unipolar RS behaviors. The high ratio of OFF/ON states over 5400 was verified with 450 reversal cycles. The current–voltage characteristics of the HZO samples showed a Schottky emission conduction mechanism in the high voltage region (V_reset_ < V < V_set_), while at the low voltage region (V < V_reset_) the ohmic contact and SCLC are suggested to be responsible for the LRS and HRS, respectively. Combined with the conductance mechanism, the RS behavior is attributed to the joule heating and the redox reaction induced by the external electron injection.
